# Fulminant infective endocarditis with toxin-negative *Corynebacterium diphtheriae* in people with substance use experiencing homelessness, England, 2024 to 2025

**DOI:** 10.2807/1560-7917.ES.2025.30.13.2500148

**Published:** 2025-04-03

**Authors:** Tara Patel, Tasnim Anwar, Eleni Mavrogiorgou, Natasa Utjesanovic, Anna Aryee, David J Litt, Joshua C. D’Aeth, Christopher Primus, Satya Das, Nikolaos Karogiannis, Karthik Paranthaman, Gayatri Amirthalingam, Rebecca Cordery

**Affiliations:** 1 UK Health Security Agency, London, United Kingdom; 2 Barts Health NHS Trust, London, United Kingdom; 3 East and North Hertfordshire NHS Trust, United Kingdom; * These authors contributed equally to this work and share first authorship.

**Keywords:** endocarditis, surveillance, diphtheria, corynebacterium

## Abstract

Between July 2024 and January 2025, five male patients in their early 20s to early 50s were confirmed with infective endocarditis associated with non-toxigenic *Corynebacterium diphtheriae* in England. Three were known to have experienced homelessness. All five used non-intravenous recreational drugs. Disease progression was rapid, four patients required surgical intervention, one died. Whole-genome sequencing and multilocus sequence type (MLST) analysis identified four individuals as ST559. Clinicians and substance use services have been alerted and enhanced surveillance implemented. A prevalence study is planned.

Non-toxigenic *Corynebacterium diphtheriae*, a Gram-positive, aerobic bacillus frequently linked to wound infections, is an uncommon cause of infective endocarditis [1,2]. This presentation has historically been associated with underlying cardiac risk factors and often a history of intravenous drug use (IVDU) [3]. While active surveillance of non-toxigenic *C. diphtheriae* is not conducted in England, an increase in clinical reports of non-toxigenic *C. diphtheriae* causing severe infective endocarditis noted by a tertiary referral endocarditis centre in early January 2025, led the United Kingdom Health Security Agency (UKHSA) to launch an investigation. Retrospective and prospective case findings were initiated. Here, we here describe the findings so far.

## Epidemiological investigation and laboratory findings

A confirmed case was defined as any person with radiological evidence (echocardiographic) of infective endocarditis, *C. diphtheriae* isolated from a sterile site and a non-toxigenic strain confirmed by the national reference laboratory at UKHSA, Colindale, London.

Initial case finding using the national diphtheria surveillance and laboratory datasets identified five cases with onset from July 2024 to January 2025, all in London and neighbouring regions, and with no more than one case per month. All were male, and the majority were young (median age: 30 years; range: early 20s to early 50s). All cases had a history of smoking or nasal insufflation of heroin, crack cocaine or powdered cocaine. Although all individuals reported no IVDU, two acknowledged past use and there were no clinical stigmata of injection.

Of the five cases, three experienced street homelessness in London. One case reported living in shared accommodation and another reported living in private housing. No clear epidemiological link between cases has been identified. Two of the individuals who experienced homelessness were found collapsed on the street.

Non-toxin producing *C*. *diphtheriae* was isolated from blood culture for all cases. Whole-genome sequencing and subsequent multilocus sequence type (MLST) analysis identified four ST559 and one ST8 isolate [4]. Core-genome MLST (cgMLST) analysis, using the *Corynebacterium* schema, revealed large distances, with 959 allelic differences separating ST559 and ST8 isolates (Figure) [5].

**Figure:**
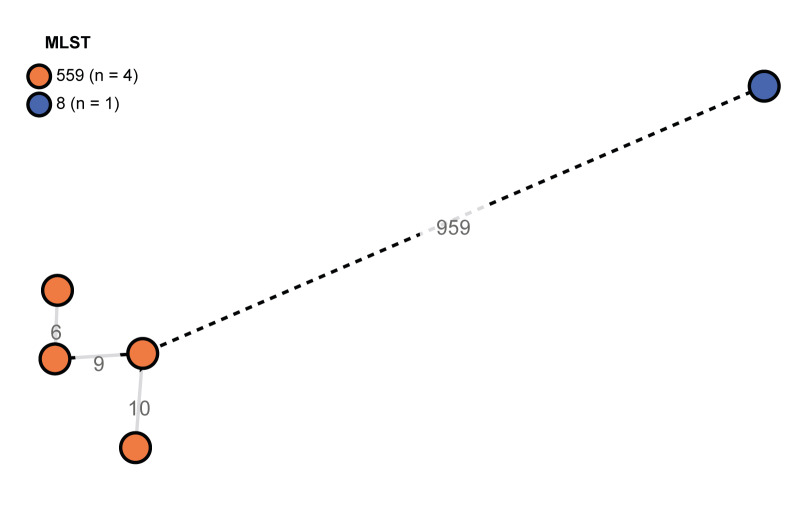
Minimum spanning tree based on cgMLST data from five *Corynebacterium* *diphtheriae* strains isolated from samples of five respective cases, with allelic distances between the strains^a^, England, July 2024−January 2025 (n = 5 strains)

cgMLST: core-genome multilocus sequence type; MLST: multilocus sequence type.

^a^ The distances are based on the number of cgMLST alleles differing between isolates [5].

Nodes are coloured by MLST of isolate, dashed line represents a shortened branch for plotting purposes. Branch labels represent the number of allele differences between isolates.

## Antibiotic treatment and clinical characteristics

Local hospital antibiotic susceptibility testing revealed that isolates from all respective individuals had benzylpenicillin minimum inhibitory concentrations (MICs) in the susceptible, increased exposure range (Table 1). Individual hospitals performed testing in line with UK Standards of Microbiological Investigations and interpreted MIC results according to the European Committee on Antimicrobial Susceptibility Testing (EUCAST) breakpoint tables for interpretation of MICs and zone diameters [6].

**Table 1:** Antibiotic treatment regimens and sensitivity profiles of confirmed cases of non-toxigenic *Corynebacterium diphtheriae* infective endocarditis, England, July 2024−January 2025 (n = 5 cases)

Case	Organism (MLST)	Sample type	Antibiotic (MIC in mg/L)	Treatment
1	ST559	Blood culture	Benzylpenicillin (0.094)	Various antibiotics first 5 days of admission (including vancomycin, ciprofloxacin, gentamicin, clindamycin) then benzylpenicillin until death (4 weeks).
2	ST559	Blood culture	Benzylpenicillin (0.094)	Amoxicillin, flucloxacillin and gentamicin for first 2 days of admission, then benzylpenicillin and gentamicin for 2 weeks until date of surgery. Post-operatively the patient received benzylpenicillin for 3 weeks and ceftriaxone for 1 week (total of 4 weeks *C. diphtheriae*-targeted treatment post-operatively). Additionally, the patient received short courses of treatment with piperacillin−tazobactam, ciprofloxacin, temocillin, amoxicillin−clavulanic acid and clindamycin for hospital-acquired pneumonia during admission.
3	ST559	Blood culture	Benzylpenicillin (0.19)	Benzylpenicillin for 6 weeks
4	ST559	Blood culture	Benzylpenicillin (0.064)	Combinations of amoxicillin, flucloxacillin, vancomycin and gentamicin during first week of admission until date of surgery. Post-operatively received benzylpenicillin for 4 weeks (with synergistic gentamicin for the first 10 days of this).
5	ST8	Blood culture	Benzylpenicillin (0.125)	Benzylpenicillin, linezolid, clarithromycin for 2 weeks, then received 4 weeks of benzylpenicillin post operatively.

MLST: multilocus sequence type.

Initial symptoms were non-specific, with two individuals describing fevers and symptoms suggestive of respiratory infection between 2 and 6 weeks before admission (Table 2). At presentation to health services, all individuals had rapid disease progression with large vegetations seen on echocardiogram. Clinical course was complicated by multiple central and/or peripheral embolic episodes despite appropriate antibiotics.

**Table 2:** Clinical characteristics and surgical summary confirmed cases of non-toxigenic *Corynebacterium diphtheriae* infective endocarditis, England, July 2024−January 2025 (n = 5 cases)

Case	Age group in years	Housing situation	Drug use	Clinical progression	Time from onset to presentation	Time from admission to surgery	Type of surgery	Outcome
1	30−34	Homelessness	Smoking: nicotine, crack/cocaine, heroin, previous IVDU	Haemoptysis, fevers, weight loss, fatigue; small hand lesion. TV IE; multiple pulmonary and septic emboli; extensive PE of the right main artery. Vegetations aTVL 4.2 × 3.45 cm, pTVL 3.54 × 1.61 cm	6 weeks	27 days	Tissue TV replacement + pulmonary thrombectomy	Failed thrombectomy. Died
2	20−24	Homelessness	Snorting: cocaine, heroin; smoking: nicotine	Found collapsed with chest pain, global ST elevation on ECG; AV and MV IE with aortic root abscess and emboli to kidneys, spleen and brain. AV largest vegetation 2.3 cm, AV NCC 1.9 × 0.4 cm, MV largest vegetation aMVL 2 × 0.6 cm	1 day – chest pain/collapse 17 days – initial presentation of wrist abscess	15 days	Mechanical AV replacement; mechanical MV replacement; aortic root patch repair	Recovered. Discharged after prolonged course of antibiotics for bilateral post-operative *Klebsiella pneumoniae* pneumonia
3	50−54	Shared accommodation	Smoking: crack cocaine, heroin	Shoulder pain, weight loss, fever; MV, AV and TV IE. MV, TV and AV vegetations: pMVL 1.5 cm × 0.3 cm, aMVL 0.7 × 0.5 cm, pTVL no quantifiable data, AV NCC 0.52 cm × 0.40 cm, LCC 0.50 cm × 0.39 cm	5 days	N/A	N/A	Discharged; under close follow-up
4	35−39	Homelessness	Smoking: crack/cocaine, heroin, previous IVDU	Found unresponsive. Cough (with green sputum), fevers; AV IE with large AV and possible small MV vegetations: AV NCC and RCC (largest 1.1 cm × 0.8 cm)	2 weeks	5 days	Tissue AV replacement	Discharged
5	20−24	Private housing	Snorting: crack; smoking: marijuana	Diarrhoea and vomiting, meningism, confusion, rash on legs; MV IE; MV vegetation pMVL 2.23 cm × 1.15 cm	4 days	14 days	Tissue MV replacement	Recovering post-surgery

aMVL: anterior mitral valve leaflet; aTVL: anterior tricuspid valve leaflet; AV: aortic valve; ECG: electrocardiogram; HAP: hospital-acquired pneumonia; IE: infective endocarditis; IVDU: intravenous drug use; LCC: left coronary cusp; MV: mitral valve; N/A: not applicable; NCC: non-coronary cusp; PE: pulmonary embolus; pTVL: posterior tricuspid valve leaflet; RCC: right coronary cusp; TV: tricuspid valve.

Four of five patients required valve replacement, with marked tissue destruction noted at the time of surgery. The mean time from admission to surgery was 15.3 days (range: 5–27 days). Clinical teams reported where infection was advanced at the time of surgery, that the clinical course was more challenging, and recovery protracted. One patient has died.

In two patients, cutaneous lesions were observed at presentation. One had almost fully healed lesions on the hand, while another had a wrist abscess a month before presentation, from which *Staphylococcus aureus* and *Streptococcus dysgalactiae* were isolated; however, the lesion was healing at the time of presentation. No other organisms were isolated from blood cultures or other specimens.

There was no significant past medical history except in one patient who had chronic hepatitis C and a recent deep vein thrombosis.

## Discussion

This report describes the first documented cluster in England of non-toxigenic *C. diphtheriae* infections, presenting as infective endocarditis, in individuals who would not generally be considered high risk, as they had no cardiac risk factors e.g. prosthetic heart valves and were not recent intravenous drug users. Case series of infective endocarditis with non-toxigenic *C*. *diphtheriae* have been reported from New Zealand (10 cases between 1994 and 2007) and Australia (7 in a single year in 1993, including one death) [7,8]. More recently a case series was reported from South Africa describing five clinically severe infective endocarditis cases with a high case fatality (80%) and associated with a novel ST of non-toxigenic *C. diphtheriae* (ST885) [9]. While these cases were all reported in 2021 from the same area, no other epidemiological links were found. One individual reported polysubstance use and only one had cardiac risk factors [9].

The severe clinical presentation and rapid progression of illness in the individuals described here are of concern, particularly in a population with poor access to healthcare, and where early diagnosis and prompt treatment are key to improved outcomes.

A key consideration is the route of bacterial entry leading to invasive infection. It is hypothesised the nasopharyngeal mucosa is the primary point of entry for the organism. However, whether this reflects colonisation of the nasopharynx with a virulent strain(s) or the contamination of products is under investigation. Indeed, the absence of pharyngeal swabs prevented determination of colonisation of the pharynx.

An individual case report, detailing a similar rapidly progressive infective endocarditis in a cocaine user, suggested direct injury to the nasal mucosa as the likely point of entry of the causative organism leading to bacteraemia, within an airway colonised with non-toxigenic *C. diphtheriae* [10].

Molecular typing of the isolates showed that most cases were infected with ST559 strains. However, historically sequencing efforts in the UK have focused on toxigenic isolates due to their association with severe clinical presentation. Given this bias, it is unclear whether the frequent detection of ST559 in these confirmed cases represents an epidemiological cluster or simply reflects ST559 as a commonly circulating strain in this population and further genomic analysis will be conducted. A study is planned to investigate the prevalence of non-toxigenic *C*. *diphtheriae* and the range of strain types circulating among people experiencing homelessness.

The UKHSA has established a national investigation to coordinate the epidemiological and public health response. Enhanced surveillance for all non-toxigenic *C. diphtheriae* isolates from sterile culture has been implemented and prompt reporting of suspected cases to the UKHSA. An enhanced surveillance questionnaire has been developed for suspected cases to systematically assess risk factors, including detail on substance use, the products and equipment employed, frequency of use and the impact of unstable housing conditions.

Raising clinical awareness is a priority to facilitate early diagnosis and treatment of cases and to support case finding. Given the severity of cases in this cluster, clinicians have been reminded to consider early echocardiography in cases with *C*. *diphtheriae* isolated from a sterile site, even in the absence of classical cardiac risk factors for infective endocarditis or a history of IVDU. Furthermore, it is imperative that all cases of infective endocarditis with non-toxigenic *C. diphtheriae* be discussed with regional Endocarditis Teams to facilitate early surgical intervention [11]. An alert has been published on the European surveillance portal for infectious diseases (Epipulse) along with direct communication with the World Health Organization to also raise awareness amongst international partners.

## Conclusions

This report raises awareness in medical and public health communities of the unusual and concerning emergence of severe infective endocarditis caused by a non-toxin producing strain of *C*. *diphtheriae*, in patients who would not be considered at high clinical risk, but who had non-intravenous substance use and, in some cases, unstable housing. Given the need for surgical intervention and challenges of ensuring consistent follow-up, the presented cluster demonstrates a particularly concerning clinical and public health issue.
